# Oral health knowledge, attitudes, and behaviors of adult patients attending a dental school hospital in Egypt: a cross-sectional study

**DOI:** 10.1038/s41598-025-29251-4

**Published:** 2025-12-05

**Authors:** Mennatollah Nagy Sharkawy, Omar Shaalan

**Affiliations:** 1 Department of Pediatric Dentistry and Dental Public Health, School of Dentistry, Newgiza University, Giza, Egypt; 2 Department of Conservative Dentistry, Faculty of Dentistry, Cairo University, Cairo, Egypt

**Keywords:** Oral health knowledge, Oral habits, Oral behaviors, Oral health attitudes, Egyptian oral health, Diseases, Health care, Medical research, Risk factors

## Abstract

Oral health is a significant contributor to public health and a basic determinant of general health. As Egypt shows high prevalence of oral diseases, the aim of this study is to assess the oral health knowledge, attitudes and behaviors (KAB) among Egyptian adults. The study also correlates the KAB of the participants with their dental habits, caries experience and sociodemographic characteristics. A cross-sectional survey with non-probability convenience sampling was performed in a dental school hospital in Egypt. The Hiroshima University - Dental Behavioral Inventory (HU-DBI) by Kawamura was used to assess the KAB, the survey also included questions about sociodemographic data and dental habits of the participants. In addition, a DMFT index was used to assess the caries experience. Continuous data was explored for normality using Kolmogrov Smirnov test and Shapiro Wilktest. Correlation between age, DMF, KAB and HU-DBI index was performed using spearman’s correlation. Association between categorical data and HU-DBI index was performed using the Chi-Square test. Significance level was set at *P* ≤ 0.05 and all tests were two tailed. The study participants demonstrated fair HU-DBI score. Knowledge (*r* = 0.70), attitudes (*r* = 0.59) and behaviors (*r* = 0.47) had strong positive correlation with the HU-DBI score. The DMFT score showed a moderate negative correlation (*r*=-0.35) with the overall HU DBI score. In addition, age showed a weak negative correlation (*r*=-0.29) with HU DBI score. The dental habits and sociodemographic characteristics showed statistically significant effect on the overall HU-DBI score, with the exception for mouth wash use, gender, health status and smoking status which showed no significant effect. Adult patients in Egypt demonstrate fair oral health knowledge and attitude and poor oral health behaviors, in addition to high caries index. We recommend planning and implementing oral health programs to enhance the oral health of adults in Egypt.

**Clinical trial number**: The protocol of the study is registered with ClinicalTrials.gov Identifier: NCT06689202.

**Supplementary Information:**

The online version contains supplementary material available at 10.1038/s41598-025-29251-4.

## Introduction

Oral health is a significant contributor to public health, and a basic determinant of an individual’s general health and quality of life^1,2^. Moreover, oral health is classified by the World Health Organization (WHO) as a major global health concern^3^. Notably, one in two adults suffer from dental decay, with oral diseases ranking among the most prevalent non-communicable diseases (NCDs)^4^. The global oral health status report shows that Egypt is among the countries with the highest prevalence of oral diseases in the Middle East and North Africa^5^.

Oral health professionals have a crucial role in influencing the behaviours of their patients and preventing oral diseases^6^. Thus, public health programs focus on prevention rather than treatment of dental diseases through modifying the lifestyle factors of the individuals such as improving their oral hygiene practices and promoting better dietary habits^7,8^. Also, effective prevention depends on enhancing oral health knowledge which is essential for positive health behaviours and better oral health outcomes^9^.

Oral health education and promotion programs have successfully improved oral health outcomes, positively influencing dental visits, attitudes, and oral hygiene practices such as brushing and flossing^10^. However, public health programs require a thorough assessment of the target population’s oral health knowledge, attitudes and behaviours. In addition, those programs must consider the sociodemographic factors of the individuals, along with their dental visiting habits which affect their oral health^11,12^.

To the best of our knowledge there is a research gap in the assessment of the oral health knowledge, attitudes and behaviours (KAB) of adult patients in Egypt. Thus, the aim of this study is to assess the KAB of adult patients attending the diagnostic center at a dental school in Egypt. The study will also assess dental habits and measure the mean number of decayed, missing, and filled Permanent Teeth (DMFT scores) to assess the participants’ caries experience. Finally, the KAB scores will be correlated with the dental habits, DMFT scores and sociodemographic variables of the participants.

## Methods

### Study design and recruitment of participants

A cross-sectional survey with non-probability convenience sampling was conducted in the diagnostic center of the School of Dentistry in one of the Egyptian universities. The research protocol was approved by the Research Ethics Committee of School of Dentistry, Newgiza University (approval date: 17 December 2024). All research was performed in accordance with the ethical principles outlined in the 2013 revision of the Declaration of Helsinki. The protocol of the study is registered with ClinicalTrials.gov Identifier: NCT06689202. The study included adult patients (18 years or older) with adequate cognitive ability who agreed to participate in the study and signed an informed consent.

#### Sample size calculation

According to the results of Al-wesabi et al. 2019^13^, the standard deviation of HU-DBI index in Egypt was 1.73. Based on the equation $\:\frac{{{Z}_{1-\alpha\:/2}}^{2}\:{SD}^{2}}{{d}^{2}}\:$by Charan et al. in 2013^14^, precision of 0.2 and by adopting an alpha (α) level of 0.05 (5%), power = 80%, the predicted sample size (n) was a total of 288 participants. The sample size was calculated using statistics kingdom sample size calculator (https://www.statskingdom.com/50_ci_sample_size.html).

### Methods of assessment

The Hiroshima University - Dental Behavioral Inventory (HU-DBI) by Kawamura was used as a validated instrument to assess oral health-related knowledge, attitudes, and behaviours (KAB) among participants (Table 1)^15,16^. The Arabic version of the HU-DBI questionnaire was validated and cross-culturally adapted, showing almost perfect reliability and validity^17^. The assessment included 20 HU-DBI items, which employed a dichotomous response format (agree/disagree) and related to oral and dental health, as well as tooth brushing habits. However, only 12 items in bold font (Questions 2, 4, 6, 8, 9, 10, 11, 12, 14, 15, 16, and 19) were used to calculate the overall KAB score. The scores ranged from 0 to 12, with higher scores indicating better oral health KAB^13,18^.

**Table 1:** The Hiroshima University-Dental behavioural inventory (HU-DBI) 20 items survey^15,16^.

No.	Question	Agree	Disagree
1	I do not worry much about visiting the dentist.		
2	**My gum tends to bleed when I brush my teeth. (D)**		
3	I worry about the colour of my teeth.		
4	**I have noticed some white sticky deposits on my teeth. (A)**		
5	I use a child sized toothbrush.		
6	**I think that I cannot help having false teeth when I am old. (D)**		
7	I am bothered by the colour of my gum.		
8	**I think my teeth are getting worse despite my daily brushing. (D)**		
9	**I brush each of my teeth carefully. (A)**		
10	**I have never been taught professionally how to brush. (D)**		
11	**I think I can clean my teeth well without using toothpaste.** (A)		
12	**I often check my teeth in a mirror after brushing. (A)**		
13	I worry about having bad breath.		
14	**It is impossible to prevent gum disease with tooth brushing alone. (D)**		
15	**I put off going to dentist until I have a toothache. (D)**		
16	**I have used a dye to see how clean my teeth are. (A)**		
17	I use a toothbrush which has hard bristles.		
18	I do not feel I have brushed well unless I brush with hard strokes.		
19	**I feel I sometimes take too much time to brush my teeth. (A)**		
20	I have had my dentist tell me that I brush very well		

The knowledge (K) index score, is derived from items 2, 8, 10, 15, and 19, the attitude (A) index score is derived from items 6, 11, and 14, and the behavior (B) index score is determined from items 4, 9, 12, and 16. One point was given for answering “Agree” to items 4, 9, 11, 12, 16, and 19, while one point was given for answering “Disagree” to items 2, 6, 8, 10, 14, and 15. Questions 1, 3, 5, 7, 13, 17, 18, and 20 are considered dummy items and are not included in the KAB score. These dummy items serve as a strategic tool in questionnaire design to enhance the quality and reliability of the collected data by mitigating biases and encouraging more accurate and authentic responses^13,18^.

The overall HU-DBI score can be interpreted as follows: a score of 0–4 (poor), 5–8 (fair), and 9–12 (good), with 12 being the maximum possible score. In addition, the Knowledge (K) index score, can be interpreted as follows: a score of 0–1 (poor), 2–3 (fair), and 4–5 (good), with 5 being the maximum possible score. Furthermore, the attitude (A) index score can be interpreted as follows: a score of 0 (poor), 1–2 (fair), and 3 (good), with 3 being the maximum possible score. Finally, the behavior (B) index score can be interpreted as follows: a score of 0–1 (poor), 2–3 (fair), and 4 (good), with 4 being the maximum possible score.

Additionally, the questionnaire included questions about sociodemographic data and dental habits to correlate these factors with other outcomes (Appendix 1). DMFT index^19^ was used to clinically determine the number of decayed (D), missed (M), filled (F) teeth (T) of the participants on the same day of the questionnaire (Appendix 2).

### Statistical analysis

The identity of the patients was kept anonymous, and the data was saved and tabulated on shared folders for back up. Statistical analysis was performed using Medcalc software, version 22 for windows (MedCalc Software Ltd, Ostend, Belgium). Continuous data were tested for normality using the Kolmogrov Smirnov and Shapiro Wilk tests and since the distribution was non-parametric, the results were presented as mean, standard deviation (SD), median, minimum and maximum. Categorical and binary data were presented as frequency and percentage. Correlation between age, DMFT, KAB and HU-DBI score was performed using spearman’s correlation. Association between categorical data and HU-DBI score was performed using the Chi-Square test. Significance level was set at *P* ≤ 0.05 and all tests were two tailed.

## Results

### Sociodemographic characteristics of the participants

Data were collected from 288 participants with a response rate of 90%. The mean age of the participants was 32.6 years ± 11.62 ranging from 17 to 72 years. As presented in Table 2 most of the participants (75%) were females while 25% were males. In addition, about half of the participants (45.8%) received higher education, while about 11% did not receive any form of education. Also, more than 60% of the participants resided in urban areas and almost half of the participants were employed (51.2%), 38.5% were unemployed or retired and the rest of the participants (10.1%) were students. Additionally, most of the participants demonstrated no systemic diseases and never smoked, however more than half of the participants (56.6%) did not have any form of insurance (private or public).

**Table 2:** Sociodemographic characteristics and dental habits of the participants.

Variable	Categories	n	%	HU-DBI	
				Median (range)	P value
Gender	Male	72	25	6 (2–10)	*P* = 0.170676
	Female	216	75	6 (1–12)	
Level of Education	None	31	10.8	5 (1–8)	*P* < 0.000001
	Basic	57	19.8	5 (2–9)	
	Middle	68	23.6	5 (2–10)	
	High	132	45.8	6 (3–12)	
Place of residence	Rural	104	36.1	5 (1–9)	*P* < 0.000001
	Urban	184	63.9	6 (2–12)	
Occupation	Agriculture & Environmental	1	0.3	4 (4–4)	*P* < 0.000001
	Arts, Media & Design	1	0.3	6 (6–6)	
	Business & Management	1	0.3	5 (5–5)	
	Education & Research	5	1.7	7 (5–9)	
	Healthcare & Medical	82	28.5	6 (3–12)	
	Hospitality & Tourism	1	0.3	5 (5–5)	
	Labour	50	17.4	5 (2–9)	
	Legal & Public Service	2	0.7	5 (3–7)	
	Service & Trade	2	0.7	5 (4–6)	
	Technology & IT	3	1	7 (4–8)	
	Student	29	10.1	7 (3–9)	
	Retired	5	1.7	6 (3–7)	
	Unemployed	106	36.8	5 (1–10)	
Health status	Healthy	234	81.2	6 (1–12)	*P* = 0.063873
	Systemic disease	54	18.8	5 (2–12)	
Smoking status	Never	234	81.2	6 (1–12)	*P* = 0.958580
	Former	18	6.2	5 (3–9)	
	Light	17	5.9	5 (3–9)	
	Heavy	19	6.6	6 (3–9)	
Insurance	No	163	56.6	5 (1–10)	*P* < 0.000001
	Private	112	38.9	7 (2–12)	
	Public	13	4.5	6 (3–9)	
Frequency of dental visits	6 months	35	12.2	7 (4–12)	*P* < 0.000001
	Annually	16	5.6	6.5 (5–9)	
	Pain	224	77.8	5 (1–10)	
	Never	13	4.5	6 (4–9)	
Reason for dental visit	Check-up	146	50.7	6 (2–12)	*P* < 0.000001
	Ongoing treatment	37	12.8	5 (1–9)	
	Pain	105	36.5	5 (2–10)	
Daily fluoride toothpaste	Yes	161	55.9	6 (2–12)	*P* < 0.000001
	No	127	44.1	5 (1–9)	
Tooth Brushing frequency	1/day	67	23.30%	6 (2–12)	*P* < 0.000001
	2/day	87	30.20%	6 (3–10)	
	> 2/day	13	4.50%	6 (3–9)	
	Rarely/never	121	42.00%	5 (1–9)	
Daily flossing	Yes	42	14.60%	7 (4–12)	*P* = 0.000011
	No	246	85.40%	5 (1–12)	
Mouthwash use frequency	1/day	16	5.60%	6 (4–9)	*P* = 0.508868
	2/day	7	2.40%	6 (2–8)	
	> 2/day	3	1.00%	6 (4–7)	
	Rarely/never	262	91.00%	6 (1–12)	

N = Number of participants, %=Percentage of participants.

### Dental habits and oral hygiene practices

Table 2 shows that most participants (77.8%) reported visiting the dental clinic only when experiencing pain. However, the remaining participants visited the dentist every six months (12.2%), annually (5.5%) or had their first dental visit on the day of the questionnaire (4.5%). In addition, on the day of the questionnaire, half of the participants (50.7%) visited the dental clinics for a dental checkup, while the remaining participants attended due to pain (36.5%) or were undergoing treatment (12.8%). More than half of the participants (55.9%) reported using fluoride toothpaste daily, however only a third (30.2%) reported brushing their teeth twice a day. On the other hand, daily flossing and mouthwash use were uncommon among the participants with 85.4% not flossing and 91% not using mouthwash.

### Correlations between KAB (HU-DBI) scores and DMFT, dental habits, and sociodemographic variables

**Fig. 1:**
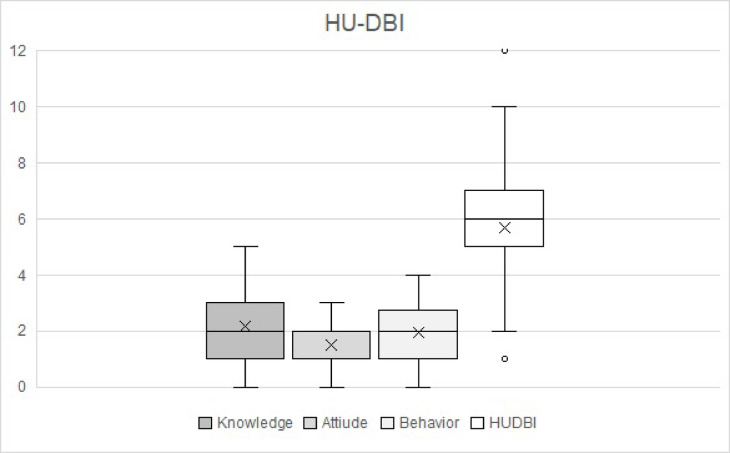
Box plot of HU-DBI total scores and subcomponents (knowledge, attitude, and behavior).

The HU-DBI scores among the participants ranged from 1 to 12, with a mean of 5.69 ± 1.80 showing a fair overall score. Additionally, the individual components knowledge and attitudes showed fair mean scores of 2.18 ± 1.09, 1.55 ± 0.96 respectively, while behaviors showed poor mean score of 1.95 ± 0.81 (Table 3). Table 3 also demonstrates the positive correlations between these components and the overall HU-DBI score, where knowledge had the strongest correlation (*r* = 0.70), followed by attitudes (*r* = 0.59) and behaviors (*r* = 0.47) all indicating strong positive correlation^20^. These correlations are illustrated in Fig. 1, which visually displays the strength of association between the HU-DBI components and the overall score. Furthermore, the mean DMFT score of the participants was 6.40 ± 5.30 indicating a high caries index^21^ and the DMFT score showed a moderate negative correlation (*r*=-0.35) with the overall HU-DBI score (Table 3).

**Table 3:** Descriptive statistics and correlations between HU-DBI and knowledge, attitude, behavior, DMFT, and age.

Numerical data	Mean ± SD	Median (range)	Correlation with HU-DBI
HU-DBI	5.69 ± 1.80	6 (1–12)	
Knowledge	2.18 ± 1.09	2 (0–5)	0.709
Attitude	1.55 ± 0.96	2 (0–3)	0.592
Behavior	1.95 ± 0.81	2 (0–4)	0.476
DMFT	6.40 ± 5.30	6 (0–25)	-0.357
Age	32.63 ± 11.62	31 (17–72)	-0.291

In terms of oral hygiene and dental habits, several factors demonstrated a statistically significant association with HU-DBI scores as presented in Table 2. Participants who reported visiting the dentist regularly every six months had higher median HU-DBI scores (7(4–12)) than those who visited annually (6.5 (5–9)), only when on pain (5(1–10)) or never (6 (4–9)). Similarly, those who visited the clinic on the day of the questionnaire for a routine checkup (50.7%) had higher median scores compared to those who came for ongoing treatment or due to pain. Additionally, participants who used fluoride toothpaste daily (55.9%) and those who practiced daily flossing (14.6%) demonstrated higher median HU-DBI scores compared to those who did not use fluoride toothpaste daily or floss respectively. However, the use of mouth wash (*P* = 0.51) did not demonstrate a statistically significant effect.

Age showed a weak negative correlation (*r* = −0.29) with the overall HU DBI score (Table 3). As shown in Table 2, most sociodemographic variables were significantly associated with HU-DBI score. Participants with higher levels of education (45.8%) had the highest median HU-DBI scores among the different education categories. Similarly, participants who lived in urban areas (63.9%) had higher median HU-DBI scores compared to those who lived in rural areas, reflecting higher knowledge, attitude and behavior towards oral health. Among the different occupation groups, participants working in education and research, as well as those in technology and IT jobs had higher median HU-DBI scores, students also showed high scores. In addition, participants with private insurance had higher median HU DBI scores compared to those with public or with no insurance suggesting better oral health awareness and behaviors. In contrast, gender (*P* = 0.17), health status (*P* = 0.06), and smoking status (*P* = 0.96) showed statistically insignificant effect on the HU-DBI score.

## Discussion

The results of the study showed that the participants demonstrated fair knowledge, attitudes and poor behaviors towards oral health, reflected by an overall fair HU-DBI score. Knowledge (*r* = 0.70), attitude (*r* = 0.59) and behavior (*r* = 0.47) had a strong positive correlation with the HU-DBI scores. Knowledge exhibited the strongest positive correlation and made the greatest contribution to the overall score. Additionally, the participants had a high caries index as indicated by their mean DMF score (6.40 ± 5.30), which is comparable to the national average DMFT score (5.5 ± 5.7) reported in a population-based survey of Egyptian adults^22^. The sociodemographic and behavioral factors showed statistically significant effect on the overall HU-DBI score, with the exception of gender, health status and smoking status and the use of mouthwash which showed no significant effect.

The fair level of oral health knowledge among the participants is consistent with previous studies among Egyptian parents^23^ and older adults^24^. In addition, consistent with our results, unsatisfactory oral health behaviors were reported among Egyptian mothers^25^ and older adults^24^. These unsatisfactory findings highlight the need for oral health education initiatives that promote preventive practices of oral diseases^26^. On the other hand, satisfactory levels of oral health knowledge and behaviors among Egyptian parents were reported in another study^27^. This discrepancy can be explained by the different background and sociodemographic characteristics of the participants in both studies.

More acceptable levels of oral health knowledge and behaviors were reported by a study conducted among adults in the United Arab Emirates (UAE)^28^.This can be explained by the lower rates of daily oral hygiene practices in our study, where only 30.2% of the participants brushed their teeth twice daily compared to 79% of the UAE study. Additionally, only 14.6% of our participants reported daily flossing compared to 45% in the other study. Furthermore, UAE is a high-income country whereas Egypt is a middle lower income country, which explains the role of the income as an enabler to oral health^29^.This observation aligns with previous research which indicates the association between lower-income levels and inadequate levels of oral health knowledge due to limited access to dental education^30^.

In addition, receiving higher levels of education was associated with higher HU-DBI scores, which aligns with the literature indicating that oral health literacy and behaviors are positively correlated with higher education levels^28,31,32^. This relationship highlights the crucial role of education in improving the oral health awareness^33^. Similarly, younger participants, particularly students, demonstrated better KAB, explaining the weak negative correlation between age and HU-DBI scores. The negative correlation suggests that younger participants tend to have higher scores. This reflects increased awareness among younger educated groups, as indicated in another study^34,35^.

The results also indicated that living in rural areas was associated with lower levels of oral health KAB, this is consistent with the results of a study conducted among pregnant Egyptian women^32^. Lower levels of KAB among participants living in rural areas can be attributed to their increased vulnerability to geographical isolation, limited access to oral health services and more financial stresses^36^. Additionally, consistent with existing literature, having health insurance was significantly associated with better oral health behaviors^28^. Most of the participants (77.8%) reported visiting the dentist only when they experienced pain. This finding aligns with another study, which suggests that such behavior may stem from lower levels of oral health awareness and financial constraints^35^.These findings highlight the need for expanded health insurance coverage to promote better oral health behaviors and increase the affordability of dental services^37^.

On the other hand, the effect of gender on oral health knowledge is statistically insignificant (*P* = 0.17), this finding is consistent with existing literature^38^. However, studies in other Arab countries, reported that females showed statistically significant higher scores of oral health knowledge^28,39,40^. Additionally, the health status and smoking status of the participants exhibited no statistically significant effect on the overall HU-DBI score. This contrasts with findings from another study reporting that non-smokers have better oral health knowledge and behaviors compared to smokers^28^.This can be due to the variable sample size, study population and the reliance of our study on self-reported data. Also, among the various oral health habits, the use of mouth wash did not have a statistically significant effect on the overall HUD-DBI score.

This study is one of the first to examine the oral health knowledge, attitudes and behaviors among a group of Egyptian adults. The study also provided insights into the correlation of these factors with the participants’ dental habits, DMFT scores and sociodemographic characteristics. Additionally, the sample included a diverse range of sociodemographic characteristics, making it representative of the population. Moreover, the study included clinical examinations, which provided an objective assessment of the participants’ oral health.

However, this study may have several limitations. First, using convenience non-probability sampling from a single institution may introduce selection bias and limit the generalizability of the results. However, data collection from a single institution was essential due to limited funding and time, which are common constraints in developing countries. Additionally, despite its limitations, convenience sampling is suitable to identify patterns and trends by providing a snapshot of the KAB of the study population. Moreover, the dental school is in Greater Cairo, a metropolitan area, which may serve as a reasonably representative sample of the Egyptian population. In addition, some subgroup sizes, such as certain occupation categories, included very few participants, which may limit the statistical validity of subgroup comparisons. Furthermore, as a cross-sectional study, it lacks the ability to establish temporality and causality; however, it serves as a valuable foundation for future studies. Lastly, the use of close-ended questions limits the ability to capture all the dimensions of oral health knowledge, attitudes, and behaviors among the participants. Self-reported data may also introduce social desirability bias, where participants may have over-reported positive oral hygiene practices and underreported negative ones.

The findings suggest designing focused oral health awareness targeting the populations with lower education levels, and those living in rural areas to tackle the observed disparities. The programs must reinforce the adoption of healthier oral habits such as daily brushing and flossing. In addition, we recommend that governmental efforts focus on expanding medical insurance coverage to vulnerable and uninsured groups. The insurance coverage should include routine dental examinations and preventive measures, which are essential for improving individuals’ oral health^41^. Furthermore, conducting multi-center studies, with larger representative samples using probability sampling, is recommended to enhance the generalizability of the results. Also, longitudinal studies are needed to assess changes in oral health knowledge, attitudes and behaviors over time. Interventional studies are also recommended to detect causal relationships and to assess the effectiveness of health promotion programs.

## Conclusion

Adult patients in Egypt demonstrate fair oral health knowledge and attitudes while their oral health behaviors are poor. In addition, the participants demonstrate high caries index. Participants with higher education levels, urban residence, regular dental visits, and positive oral hygiene practices such as daily use of fluoride toothpaste and flossing showed higher HU-DBI scores, implying better oral health knowledge, attitudes, and behaviors. These findings indicate a substantial need for coordinated oral health promotion programs targeting different population segments.

## Supplementary Information

Below is the link to the electronic supplementary material.

Supplementary Material 1

## Data Availability

The datasets generated and/or analysed during the current study are not publicly available due to participant consent limiting data use to research purposes under conditions that ensure anonymity. Public sharing may compromise confidentiality and privacy. However, the data are available from the corresponding author on reasonable request.

## Abbreviations

HU-DBI: The Hiroshima University–Dental Behavioral Inventory
DMFT: Decayed, missing and filled teeth
KAB: Knowledge, attitude and behavior
