# Supplementary material for: Oral health knowledge, attitudes, and behaviors of adult patients attending a dental school hospital in Egypt: a cross-sectional study
Source: Sci Rep. 2025 Dec 5;15:43274. doi: 10.1038/s41598-025-29251-4 (PMC12686475; doi:10.1038/s41598-025-29251-4)
Supplement: Supplementary file 1 — Supplementary Material 1 [file 41598_2025_29251_MOESM1_ESM.docx]

**Appendix 1**

Sociodemographic Data and Dental Habits

| **Variables** | **Answer** |
| --- | --- |
| **Gender** | - Male - Female |
| **Age** | - Years |
| **Level of Education** | - None - Basic - Middle - High |
| **Smoking status** | - Never - Former - Light smoker - Heavy smoker |
| **Daily fluoride toothpaste** | - Yes - No |
| **Daily flossing** | - Yes - No |
| **Tooth brushing frequency** | - 1/day - 2/day - >2/day - Rarely/Never |
| **Mouthwash use frequency** | - 1/day - 2/day - >2/day - Rarely/Never |
| **Frequency of dental visits** | - 6 months - Annually - Biennially - Pain |
| **Reason of dental visits** | - Check-up - Ongoing treatment - Pain |
| **Occupation** | - Healthcare & Medical - Education & Research - Business & Management - Technology & IT - Engineering & Architecture - Arts, Media & Design - Service & Trade - Legal & Public Service - Finance & Insurance - Hospitality & Tourism - Agriculture & Environmental - Construction & Real Estate - Transportation & Logistics - Student - Retired - Unemployed |
| **Place of residence** | - Urban - Rural |
| **Health insurance** | - Public - Private - None |
| **Health status** | - Healthy - Systemic disease - Disabled |

**Appendix 2**

Decayed (D) teeth =

Missed (M) teeth =

Filled (F) teeth =

DMFT Score = D+M+F
